# Supplementary material for: Are carbon emissions trading and green financial instruments synergistic? -Comprehensive quantitative research based on content analysis
Source: PLoS One. 2024 Mar 7;19(3):e0298601. doi: 10.1371/journal.pone.0298601 (PMC10919601; doi:10.1371/journal.pone.0298601)
Supplement: S1 Data — (ZIP) [file pone.0298601.s001.zip › Data/地级市Z_F工作报告.zip!/╔┬╬≈/╙▄┴╓╩╨/└┤╫╘╣1⁄2╓┌║┼í╛┬φ┐╦╩2╛▌═°í┐_2016.docx]

各位代表：

现在，我代表市人民政府向大会报告工作，请予审议，并请市政协委员和列席人员提出意见。

一、2015年和“十二五”时期工作回顾

2015年是榆林发展极为艰难的一年。面对能化产品价格持续下跌、固定资产投资放缓、民间借贷危机发酵等前所未有的困难和考验，全市上下认真贯彻落实中省系列决策部署，凝心聚力，攻坚克难，统筹推进稳增长、促改革、调结构、惠民生、防风险各项工作，经济发展稳中趋好，社会大局和谐稳定。实现地区生产总值2621.3亿元，增长4.3%；完成地方财政收入295.6亿元，增长13.8%；城乡居民收入达到32078元、10636元，分别增长8.1%和9.3%；实现社会消费品零售总额396.4亿元，增长5.8%。一年来，我们主要做了以下几方面工作：

（一）抓住关键环节，力促经济企稳回升。针对一季度经济增速“断崖式”下跌局面，及时实施新增库存储备贴息、清理涉企收费、企业降本增效等一系列稳增长政策措施，经济运行稳步回升，全年实现规模以上工业增加值1562亿元，增长4.2%，总量位居全省第一。精准拓展能化产品市场，开展各类产销对接活动15场，工业品产销率达96.5%。持续强化品牌建设，“榆林煤”成功注册产地证明商标，“榆林山地苹果”和“榆林马铃薯”获得国家地理标志保护产品认证。多渠道筹措建设资金，100个重点项目完成投资382.3亿元，签约PPP项目协议13个，引进社会资本10.4亿元；争取棚户区改造资金14.3亿元，落实四批专项建设基金19.8亿元。积极破解中小微企业融资难题，组织多场政银企对接会，落实各类贷款293亿元；通海绒业、德林荣泽两家企业在新三板挂牌，实现我市企业上市零的突破，非公经济实现增加值1074.8亿元，占全市GDP比重达41%。

（二）紧扣提质增效，加快产业转型升级。科技创新取得重大突破，成立煤化工产业技术研发中心，建成榆林高科企业孵化中心，组建红枣、马铃薯两个技术创新战略联盟，煤油共炼成套工艺、低阶粉煤回转热解工艺等6项技术达到世界领先水平，形成全球最大的基因编辑绒山羊种群。能化产业转型成效显著，未来能源煤制油、神华甲醇下游深加工等一批资源深度转化项目建成投产，新增烯烃产量100万吨、甲醇25万吨。新能源产业发展势头强劲，累计建成装机241万千瓦，增长52.5%，全年上网发电32.4亿度，增长50.9%。大数据、互联网等新业态加快发展，智能通、北斗星、乡土中国等企业落户榆林，建成西北地区首个专业快递园区。文化旅游产业持续壮大，统万城、高家堡等重点景区项目顺利推进，乡村游、自驾游、近郊游加速兴起，全年接待游客1990万人次，实现旅游综合收入107.5亿元，增长12.8%。

（三）立足强基固本，推进现代农业发展。积极培育新型经营主体，新增农业龙头企业82家、农民合作社1899家、新型职业农民1538人。加快推动农业适度规模经营，流转土地5.5万亩，新增家庭农牧场507家，定边县成为全国马铃薯种植面积六大县之一，米脂县建成全市首个国家级苹果标准园。加快推进现代畜牧业标准化体系建设，羊子、生猪、肉牛等畜禽饲养规模稳定，羊绒产量占到全省的90％。加强农产品质量监管，成为全国首个农产品质量安全追溯系统整市推进的地级市。不断夯实农业发展基础，实施农村饮水安全项目900个，新建通村公路2700公里，新修改造基本农田9.9万亩，新建加固维修淤地坝876座。粮食总产量达142.9万吨，完成农业总产值250.1亿元，增长4.9%。

（四）坚持以人为本，加快推进新型城镇化。启动第五版城市总规修编，完成城市开发边界划定工作。中心城区实施各类市政项目74个，完成投资10.2亿元，环城北路西段、德静东路等项目建成通车，沙河公园、榆溪河生态长廊一期建成开放，城区绿化覆盖率达37.7%。县城和重点镇建设成效显著，横山撤县设区获国务院批准，大柳塔镇列为全省镇级市培育试点，11个县城基础设施建设完成投资21.7亿元，城乡公共服务设施不断完善。振南工作成效显著，41个重点项目完成投资6.5亿元，一批学校、医院、道路、产业项目加快建设，南部六县公共服务水平和可持续发展能力进一步提高。

（五）补齐发展短板，提升要素保障能力。内畅外联的综合交通体系不断完善，神佳高速、店红一级、大石一级公路建成通车，沿黄公路二期全线贯通，改造县乡公路203公里，启动实施210国道榆林至陕蒙界、204省道榆阳至靖边和米脂、绥德、清涧三县过境线改建等一批道路项目。电力外送通道建设快速推进，神府至河北南网500千伏第二回路项目进展顺利，榆横至山东1000千伏特高压输变电工程开工建设。水源保障能力显著提升，王圪堵水库枢纽项目建成投运，黄河多点引水工程加快推进。生态环境持续改善，实施“生态建设五年行动计划”，绿化造林80.5万亩，中心城区禁煤范围扩大到255平方公里，空气自动监测站点实现市县区全覆盖，榆林城区空气质量好于二级以上天数达到286天，居全省前列。

（六）突出问题导向，深入推进改革开放。“多规合一”试点改革成效显著，获得中央改革办充分肯定，为全国市级改革提供了“榆林经验”。行政审批制度改革深入推进，承接中省下放行政审批事项126项，精简行政审批事项79项。镇村综合改革顺利完成，减少乡镇20个，撤并建制村1265个。县以下机关公务员职务与职级并行制度稳步推进，党政机关公车改革全面完成。不动产统一登记工作走在全省前列。商事制度改革成效显著，“三证合一”“一照一码”全面实施，新增各类市场主体40872户，增长20.8%。文化体制改革深入推进，整合成立市民间艺术研究院和市戏曲研究院。对外开放水平不断提升，榆林海关正式开关，进出口总额达到3226万美元，招商引资到位资金648亿元。区域合作迈上新台阶，成功举办第三届呼包银榆经济区市长联席会议，签订五项多边合作协议。

（七）强化兜底补短，着力保障改善民生。在财政收支矛盾凸显的情况下，全面落实“两个80%”硬指标，民生投入达到375亿元，增长8.4%。社会各项事业加快发展。实施标准化学校建设和薄弱学校改造工程，推行二期学前教育三年行动计划，实施义务教育学校阳光招生和均衡编班政策，全市高考二本上线万人比居全省前列。市二院完成新址搬迁，市一院绥德院区建成投用，分级诊疗制度全面推开，城镇居民大病医疗保险工作有序推进，新农合参合率达99.9%。实施体育惠民工程392个，政府购买公共文化演出881场，《大漠红柳》《统万雄风》《杨家城传奇》列为省上重点打造剧目。社会保障体系不断完善。发放小额担保贷款5.2亿元，新增城镇就业2.9万人，转移农村劳动力75万人。城乡低保标准提高到485元/月和2500元/年。开工建设保障性住房46526套，建成24483套。投入各类扶贫资金10.8亿元，16.03万人实现精准脱贫。安全生产形势持续好转，亿元GDP事故死亡率下降32.4%。依法妥善处置非法集资、商品房退购等突出问题，信访总量下降60.5%。

（八）践行“三严三实”，全面加强政府自身建设。认真落实党风廉政建设责任制，深入开展“三严三实”专题教育，强化行政监察和审计监督，推行财政预决算公开，市本级“三公经费”下降13%。自觉接受人大依法监督、政协民主监督和社会舆论监督，人大代表建议、政协委员提案办复率均达100%。全面实行政务公开，组织开展“公民代表进政府”系列活动，启动市县区政务服务中心标准化建设，政务服务水平走在全省前列。与此同时，人口计生、民族宗教、扶残助残、人防、老龄、双拥、档案和地方志等工作都取得了新成效。

各位代表，一年来，面对多重困难和压力，我们在市委的坚强领导下，和全市广大干部群众一道，迎接挑战，攻坚克难，做了大量工作，付出了艰辛努力，除地区生产总值、固定资产投资等指标低于预期外，经济社会仍然保持了稳健发展态势，实现了“十二五”的圆满收官！

各位代表，回顾过去五年的工作，全市人民守望相助、务实拼搏，战胜各种风险挑战，取得巨大发展成就，为“十三五”时期在全省率先全面建成小康社会奠定了坚实基础。

五年来，经济实力大幅跃升。地区生产总值年均增长9.8%，总量连续五年位居全省第二；人均GDP突破1万美元，位居全省第一；地方财政收入实现翻番，年均增长18.7%；社会消费品零售总额实现翻番，年均增长13.2%；五年累计完成固定资产投资8009亿元，是“十一五”完成投资总量的2.4倍。特别是工业经济规模由1882亿元增加到3121亿元，形成煤电、兰炭、煤制油、甲醇、烯烃、铝镁材料等一批基地级的产业集群，榆林先后被纳入呼包银榆经济区发展规划、陕甘宁革命老区振兴规划等7个国家级规划，国家能源化工基地地位进一步凸显。

五年来，结构调整加速推进。三次产业结构由5.3∶68.6∶26.1演进为5.5∶62.5∶32，第三产业增加值占比提高5.9个百分点。“榆林版煤制油”、百万吨煤间接液化、世界首套万吨级甲醇制芳烃等一大批具有世界领先水平的装置成功运行，能源化工基地核心竞争力国内领先。光伏、风电装机容量占到全省的89%，入选全国首批新能源示范创建城市。全社会科技研发投入增长5倍，科技对经济增长的贡献率达到45%以上，榆林高新区、榆神工业区、榆林现代农业科技示范区升级为国家级园区。创造5类54项农作物全国高产纪录，成为全国山羊绒产量第一大市、非牧区养羊第一大市和全国马铃薯生产第三大市，建成陕西重要的现代特色农业生产基地。

五年来，发展环境显著改善。榆阳机场国内航线达到29条，年旅客吞吐量149万人次，成为西北第一支线机场。形成以太中银、包西、神朔等干线铁路为主动脉的铁路运输网络，吴堡、子洲、横山、定边和靖边5个县结束没有铁路的历史。高速公路总里程突破1000公里，位居全省第一；县乡油路和通村公路实现全覆盖。实施王圪堵水库等一批水利枢纽工程，新增供水能力2.8亿立方米。新增造林面积306.7万亩，建成绿色长廊1600多公里，林木覆盖率提高了3个百分点，能耗及化学需氧量等5项约束性指标全面下降，保障发展的能力持续提升。

五年来，城乡面貌焕然一新。中心城区建设累计完成投资120亿元，是“十一五”时期的4倍。建成区面积翻了一番，便捷通畅的路网体系基本形成，居民人均公共绿地面积由6.7平方米提高到16.7平方米，成功创建国家卫生城市、省级环保模范城市、省级园林城市和省级文明城市，城市品位显著提升。神木、府谷、靖边三县建成全国卫生县城，省级卫生县城实现全覆盖。“双十二”试点工程完成投资15亿元，城乡基础设施和公共服务水平持续改善，城镇化率达55%，较“十一五”末提高了7.6个百分点，区域城乡统筹发展水平迈上新台阶。

五年来，民生福祉持续提升。民生投入累计达到1690亿元，城镇居民人均可支配收入是“十一五”末的1.8倍，农民人均纯收入实现翻番。扶持创业3.7万人，新增城镇就业13.2万人。率先在全省实现15年免费教育，中心城区新建、改扩建各类学校42所，教育惠民水平全省领先。城镇医保、新农合保障标准高于全省平均水平，中心城区医院床位实现翻番，医改工作走在全国前列。市本级财政投入扶贫资金36.1亿元，城乡低保、城乡养老等保障水平持续提升，减少贫困人口60.1万人。

各位代表，今天的榆林，经济实力更加雄厚，基础条件更加完善，干部群众思想更加解放。五年奋斗积累的经验弥足珍贵。我们深刻体会到：必须牢固树立发展意识，善于通过推动发展来解决前进中的困难和问题；必须牢固树立机遇意识，善于把中省决策部署转化为推动转型发展的强大动力；必须牢固树立创新意识，善于向改革要活力、向创新要动力、向开放要空间；必须牢固树立以人民为中心的思想，善于让发展成果更多更公平惠及全市人民。

各位代表，五年的发展历程充满艰辛，五年的发展成果令人振奋。这是省委、省政府和市委坚强领导的结果，是市人大、市政协鼎力支持的结果，是全市人民团结拼搏的结果！在此，我代表榆林市人民政府，向全市广大干部群众，向驻榆部队指战员、武警官兵、公安干警，向所有关心、支持和参与榆林建设发展的社会各界人士，致以崇高的敬意和衷心的感谢！

在肯定成绩的同时，我们也清醒地认识到，我市经济社会发展还存在不少困难和问题。主要表现在：传统产业增长动力不足，新兴产业短期内难以形成有效支撑，稳增长压力持续加大；经济结构不优，产业低端化、产品初级化特征明显，转型升级任务十分艰巨；改革开放相对滞后，创新引领和人才支撑作用亟待提高；生态环境依然脆弱，社会事业欠账较多，各类瓶颈制约依然突出；金融、民间借贷、房地产市场、政府性债务等领域风险相互交织，防风险的任务十分繁重；政府职能转变还不够到位，解决不敢担当、为官不为等问题更为迫切。对于这些问题，我们将采取有力措施，认真加以解决。

二、“十三五”时期经济社会发展主要目标任务和重大举措

“十三五”时期，是全面建成小康社会的决胜阶段，也是我市经济社会转型发展的关键时期。当前，制约发展的不确定、不稳定因素较多，尤其是受国际能源价格持续下跌影响，我市经济稳健发展面临严峻挑战。同时也要看到，我国以煤为主的能源消费格局在较长时期内难以根本改变，作为资源成长型城市，榆林仍处于经济发展周期的上升阶段。我们有良好的发展基础。经过多年不懈奋斗，我市经济硬实力、发展软实力、综合竞争力迈上新台阶；特别是我们抓住市场倒逼机遇，上马了一大批资源综合利用项目，我市产业基础、产业规模、产业布局、产业形态等方面的比较优势逐步凸显，有利于我们在日趋激烈的市场竞争中赢得先机。我们有巨大的发展空间，我市在精细化工、特色农业、旅游文化、县域经济、统筹城乡等方面取得初步突破，进一步加快发展的潜力巨大、前景广阔；特别是新一轮科技和产业革命，将催生一批新产业、新模式、新业态，有利于我们承接产业转移、延伸产业链条、壮大新兴产业。我们有难得的政策机遇，国家加快推进“一带一路”、“互联网+”、脱贫攻坚等重大战略，省上提出建设创新型省份，明确支持陕北转型持续发展，有利于我们进一步争取中省支持；特别是中央加快供给侧结构性改革，推进“三去一降一补”，为我们解决经济发展中存在的一系列深层次矛盾和问题提供了重大机遇。我们一定要统一思想、坚定信心、奋发有为，持续不断破解发展难题、增强发展动力、补齐发展短板、厚植发展优势，奋力开创榆林经济社会发展新局面！

“十三五”时期，我们工作的指导思想是：全面贯彻党的十八大及十八届三中、四中、五中全会和习近平总书记系列重要讲话精神，牢固树立“五大发展理念”，认真落实“五位一体”总体布局和“四个全面”战略布局，始终坚持以人民为中心的发展思想，深入实施“创新引领、主动转型”战略，全面落实供给侧结构性改革要求，着力推进“一中心两基地”建设，加快形成引领经济发展新常态的体制机制和发展方式，不断提高经济发展的质量和效益，努力打造全国资源型城市转型发展先行区、陕甘宁革命老区统筹城乡试验区、西北地区生态文明建设示范区和中国内陆开放开发战略新高地。

我们的奋斗目标是：2017年率先完成脱贫攻坚任务，2019年率先全面建成小康社会。到2020年，地区生产总值达到4500亿元，年均增长6%；地方财政收入达到320亿元；城乡居民收入分别达到39000元和14000元。

（一）坚持创新发展，加快经济转型升级。大力实施创新驱动战略，将创新贯穿到经济社会发展各领域、全过程，培育和建设一批实验研究平台，突破和掌握一批关键核心技术，全力构筑发展新优势；健全完善大众创业、万众创新体制机制，促进新技术、新产业、新业态蓬勃发展，加快培育发展新动力。实施工业经济转型升级三年行动计划，建设高端低碳的现代产业体系，打造国家清洁能源输出基地、世界一流的煤化工基地、国内领先的精细化工原料生产基地和西北地区重要的战略性新兴产业基地。到2020年，科技对经济增长的贡献率达到50%以上，煤炭就地转化率提高到50%。

（二）坚持协调发展，构建协同发展格局。落实主体功能区战略，划定生态、农业、城镇空间，加快打造长城沿线城镇发展轴、沿黄生态经济带和无定河生态廊道。推进以人为核心的新型城镇化，围绕“城区、园区、景区、社区”建设，实施基础设施提升工程，加快神木、靖边、绥德撤县设市，佳县撤县设区，集中力量建设一批人口过万、产值过亿的中心镇，扎实推进美丽宜居乡村建设。继续组织实施振兴南部战略，形成区域协同发展新格局。到2020年，城镇化水平达到60%左右，中心城区建成中国宜居城市，南部六县经济总量实现翻番。

（三）坚持绿色发展，建设美丽生态家园。把生态建设作为立市之本，严格划定并执行各类生态红线，启动实施十大生态提升工程，深入推进煤炭清洁高效利用和循环低碳发展，积极推进矿区生态修复，启动实施重要河道综合整治。到2020年，绿色生产方式和生活方式深入人心，主要污染物排放完成目标任务，万元GDP能耗降低10%。中心城区建成国家环保模范城市，林木覆盖率提高3个百分点，主要河流及重点水库水质达标率在90%以上，让榆林的天更蓝、地更绿、水更清。

（四）坚持开放发展，打造内陆开放高地。主动融入国家“一带一路”战略，积极参与西安丝绸之路经济带新起点建设，打造榆林国际煤炭暨能化装备技术博览会品牌，推动跨区域口岸协作，拓展“榆林好产品”海外市场，打造陕西向北开放的“门户”。深化与呼包银榆经济区城市协作，加强与京津冀、长三角、珠三角经济圈的联系，主动承接东部沿海地区产业转移。到2020年，进出口总额力争突破1亿美元，招商引资总额占到全社会固定资产投资的40%。

（五）坚持共享发展，率先实现全面小康。精心组织实施好重点民生工程，坚决打赢脱贫攻坚战，确保到2017年，28.15万贫困人口全部脱贫，8个贫困县全部摘帽。巩固提升十五年免费教育，教育资源配置更加合理，基础教育实现均衡发展。医疗卫生、社会保障等公共服务体系进一步完善，每县建成一所二级甲等医院，基本社会保险覆盖率达到95%以上。多渠道增加城乡居民收入，年均增长7%以上。推进法治榆林建设，织好民生安全保障网，让人民群众有更多获得感。

各位代表，全市人民对幸福生活的向往和期待，就是我们的追求和目标。我们坚信，通过全市广大干部群众的团结拼搏和不懈努力，“十三五”的宏伟蓝图一定能够变成美好现实，一个经济更加繁荣、社会更加和谐、环境更加优美、人民更加幸福的新榆林必将崛起在西部大地！

三、2016年的主要工作任务

今年是“十三五”的开局之年，也是新一届政府履职尽责的起步之年，做好全年工作至关重要。我们将认真学习贯彻中省会议精神，全面落实市委三届七次全会的工作部署，坚持稳中求进工作总基调，落实推进供给侧结构性改革要求，统筹推进稳增长、调结构、惠民生、防风险各项工作，努力推动经济社会平稳健康发展，为“十三五”发展开好局、起好步。主要预期目标是：地区生产总值增长6.5%左右，全社会固定资产投资增长6%，地方财政收入增长3.9%，城乡居民收入分别增长6.5%和8%，社会消费品零售总额增长10%，城镇登记失业率控制在3.9%以内，全面完成省上下达的节能减排任务。

（一）推进供给侧结构性改革，促进经济平稳健康发展。

综合施策稳定实体经济。认真贯彻落实中省稳增长重大部署，制定促进实体经济健康发展政策。加强对经济运行的分析研判和监测调度，全力稳定实体经济的生产经营。实施质量强市和品牌引领战略，抓好“榆林煤”中国驰名商标申报和“榆林兰炭”地标产品认证工作，持续打造“榆林好产品”品牌，不断提升市场竞争力。继续推行企业分类管理，扎实开展“降本增效”专项行动，切实减轻企业负担。毫不动摇支持民营经济发展，放宽电力、交通、市政公用等领域市场准入，鼓励民营经济参与国有企业改革，发展混合所有制经济；继续强化政银企对接，支持民营企业技术改造、整合重组，抓好企业上市培育工作。落实中省去产能政策，推进60万吨以下煤矿整合重组，强化对环保不达标、安全有隐患企业的治理，引导硅铁、小火电等“三高一低”落后产能有序退出。

全力以赴扩大有效投资。扎实推进90个市级重点项目，力争完成投资400亿元。加快推进准朔、蒙华、靖神铁路和小纪汗煤矿、孤庙、郭神3条运煤专线建设，启动包西高铁榆林段前期工作。推进榆靖高速互通立交、沿黄公路提升、榆阳至靖边等9个续建项目建设，建成绥德、清涧县城过境公路，启动吴定、绥延高速建设，实施横山、子洲等6条县城过境公路项目。加快定边、清涧黄河引水及榆佳工业园区供水项目建设，建成横山县城供水工程。加快推进榆横到山东特高压输电通道建设，开工建设陕北至关中第二输电通道。实施光纤到楼入户和下乡进村工程，普及城区公共无线网络，建设“宽带榆林”。认真做好项目策划储备，积极争取国家专项建设基金和各类政策性贷款，力争落地建设一批新的重大项目。

千方百计释放消费潜力。优化消费环境，逐步引导埠外消费回流。引进全国知名零售企业，打造环境一流的城市商业综合体，力争华润万家榆林高新店开业运营。支持传统商贸企业搭建网络交易平台，实现线上线下互动发展。加快快递物流业发展，建设神府、榆阳、绥德、定靖4个电商物流园区和8个县级专业快递园区。启动基层供销社三年提升行动计划，促进农村电商健康发展，解决农民群众的“买难卖难”问题。积极培育消费新热点，拓展健康、养老、家政、文化、体育等消费新领域，壮大网络信息、智能家居、个性时尚等新兴消费。推进房地产去库存，推行棚改货币化安置，鼓励农民工进城购房落户，促进房地产市场健康发展。落实带薪休假制度，鼓励有条件的单位开展2.5天休假试点，激活假日消费。

（二）调整优化产业结构，提升经济发展的质量和效益。

加快发展高端能化产业。推进榆能横山、国华锦界、华电红墩界等7个煤电一体化项目建设，确保清水川煤电二期项目建成并网发电，力争煤电装机达到1500万千瓦。扎实推进延长石油煤炭清洁综合利用、陕煤煤炭分质清洁转化等重大项目建设，启动富油50万吨环烷基油项目，力争华电煤基芳烃项目开工建设、中煤榆林煤炭深加工项目完成核准、兖矿煤制油二期项目纳入国家相关规划，确保全年新增煤制油70万吨、烯烃60万吨。大力发展油气深加工产业，开工建设定边50万吨沥青、200万吨油气转化综合利用和榆炼成品油库等项目，打造千万吨级炼化基地。围绕甲醇、烯烃、煤焦油、聚氯乙烯等初级能化产品，全力培育引进一批精细化工产业项目，着力发展烯烃下游、芳烃下游、聚酯下游、氯碱下游、铝镁材料等高附加值产业链，全面提升能源化工基地发展水平。

培育壮大新兴产业。扎实推进华电100兆瓦风电、隆源200兆瓦光伏等新能源项目建设，大力发展风光互补、农光互补和分布式光伏电站，新增新能源装机80万千瓦；加快吉山梁、公布井等汇集升压站及外送配套工程建设，缓解新能源电力输出瓶颈；抓好陕西有色多晶硅、定边正信光伏组件等项目，推进光伏产业全产业链发展。大力发展战略性新兴产业，引进落地一批蓝宝石玻璃、激光晶体项目，积极推进碳化硅、石墨烯等新材料的研发应用，加快发展黄芪、麻黄等生物制药产业。加强与知名互联网企业的合作，布局一批云计算、大数据、物联网等前沿技术项目，建设区域性数据基地。

改造提升传统产业。推进兰炭产业转型升级，支持兰炭绿色生产、煤焦油深加工等关键技术攻关，推动大型煤基热解项目落地。抓好银丰陶瓷二期、绥德臻梦铝镁合金等项目建设，培育壮大装备建材产业。支持羊老大、七只羊、羊中王等品牌做大做强，打造全国最大羊毛防寒服生产基地。加快红枣多糖技术工业化，推进蓖麻、花卉、沙蒿、长柄扁桃等生物质提取，开展绿豆、玉米、大豆、猪牛羊副产品生物技术综合利用研发，让传统产业焕发出新的生机和活力。

大力发展旅游文化产业。实施全域旅游战略，召开全市旅游文化产业发展大会，系统谋划旅游文化产业发展路径，出台促进旅游文化产业发展的政策措施。加快石峁遗址的有序发掘和保护，推进统万城国家考古遗址公园等重点景区建设，加强明长城抢救性保护，做好镇北台、红石峡、榆林古城一体化保护开发的前期工作，抓好红碱淖、白云山5A级景区创建工作，策划中国盐湖游乐园项目。联手延安、吕梁、鄂尔多斯等周边地市，打造长城边塞游、黄河风情游、转战陕北红色体验游等旅游线路，支持佳县创建国家全域旅游示范县，神木和绥德创建省级旅游示范县，努力建设西北地区特色旅游目的地。实施精品影视剧目创作工程，开拍《平凡的世界（二）》等系列影视剧目，打造陕北民歌、榆林说书、榆林剪纸等一批榆林文化符号，提升榆林文化软实力。

（三）坚持以人为本，统筹城乡区域协调发展。

提高城市治理能力。强化规划立市理念，完成榆林市第五版城市总规修编工作，启动中心城区“一张图”工程，推进海绵城市、地下综合管廊等规划编制工作。加强中心城区基础设施建设，重点实施长城路南延、榆横五路与榆溪大道连接通道等18个路网项目，加快推进环城北路西段供热、西沙水厂、东郊配气站等一批市政工程，实施榆溪河生态长廊南北段和大墩梁尤家峁自然生态保护区一期工程。围绕榆溪河、榆阳河治理和老城区保护推进棚户区改造，实施崇文雅苑、驼城家园、滨水西岸三个棚改项目。深化城市管理体制改革，推进“智慧城管”建设，加强对城乡结合部、城中村违建行为的管控，扎实推进“一巩固五创建”工作。加强城市社区建设，认真做好社区达标改扩建工作，不断提升管理服务水平。支持各县区争取国家专项建设基金，加快实施一批打基础、利长远的公共服务和基础设施项目，不断增强县城的承载能力和辐射带动能力。

推进“美丽镇村”建设。支持榆阳、神木、横山列为全省新型城镇化试点县区，推进大柳塔镇级市培育试点改革，加快锦界、东坑2个省级重点示范镇和高家堡、波罗、名州3个省级文化旅游名镇建设，巩固提升市级“双十二”试点成果。继续加大重点集镇建设，支持各县区打造一批产城融合、功能齐备的特色小镇；深化“美丽乡村”建设行动，按照城乡基础设施和基本公共服务均等化要求，扎实推进“五覆盖六到户”工程，建设一批宜居、宜业、宜游的“美丽乡村”。

促进区域协调发展。以壮大县域经济为切入点，立足资源禀赋和区域特点，着力构建各具特色、优势互补的协同发展格局。支持北部县区转型发展。推进资源清洁高效利用，做大做强能化产业。培育壮大战略性新兴产业，构建多元发展、多极支撑的现代产业体系，切实增强可持续发展能力。支持南部六县加快发展。完善四位一体的振南机制，统筹使用各类帮扶资金，加快布局一批带动能力强的造血项目，持续做大经济总量。支持各类园区创新发展。发挥好榆林高新区、榆神工业区、农业科技示范区的引领示范作用，积极推进县级园区管理创新、服务创新和模式创新，着力提升园区服务水平和承载能力，强化园区对县域经济的支撑作用。

（四）抓好“三农”工作，夯实全面建成小康社会的基础。

抓好精准扶贫工作。认真落实全市脱贫攻坚会议工作部署，聚焦“脱贫”“摘帽”两大任务，带着责任、带着感情，真扶贫、扶真贫，全力以赴打好脱贫攻坚战。扎实推进移民搬迁工作，切实抓好贫困户危房改造，全年完成1.3万户、4.5万人的移民搬迁任务。鼓励县区加大资金捆绑整合力度，做好基础设施建设、增收产业培育等工作，发展一批旅游、分布式光伏、农产品深加工等产业项目，提升贫困地区、贫困人口的自我发展能力。实行农村低保标准与脱贫标准两线合一，建立多层次、全覆盖的贫困家庭在校生助学体系，全面推开重特大疾病救助，落实好社保兜底政策，年内确保16万人实现精准稳定脱贫，478个村退出脱贫村序列，定边县实现整体脱贫。

推进现代特色农业发展。加快农业结构调整，稳步推进以玉米、马铃薯、小杂粮为重点的标准化生产基地建设。积极培育适度规模经营的新型农业经营主体，开展好“十大农业龙头明星企业”评选活动，粮食总产量稳定在140万吨左右。大力发展林果产业，支持山地苹果、核桃、红枣等特色优势产业发展壮大，新建优质山地苹果5.5万亩和特色时令水果1.5万亩。推进畜牧业提质增效，稳定畜禽饲养规模，加大粮经饲草统筹，推动农林牧一体发展。培育“粮改饲”示范村5个、示范户100个，推广种植饲料作物50万亩、人工种草100万亩。加快发展农产品加工产业和休闲观光农业，扎实推进定边10万吨马铃薯主食加工项目，新建规模化屠宰加工厂3个；规范提升10个“一村一品”精品示范村，打造30个市级休闲农业示范点，推动一二三产业融合发展，让更多农户分享全产业链价值收益。

改善农村基础设施。改造县乡公路200公里，新建通村公路1000公里，加快具备条件的乡镇和建制村通客车。启动新一轮农村电网改造工程，解决9万农村人口安全饮水问题，新修改造基本农田7.6万亩，新建加固淤地坝850座，完成水土流失治理1130平方公里，不断夯实农业发展基础。

（五）扎实推进改革创新，增强经济发展动力和活力。

深入实施创新驱动战略。推进重大核心技术攻关，支持企业加大科研投入，推动煤油气绿色开采、煤炭分质利用、农业抗旱保收、畜禽良种选育等领域的关键技术取得突破。抓好产学研协同创新，支持各类园区和重点企业建设一批研发机构和中试基地，促进更多科技成果在我市转化落地。推进大众创业、万众创新，用足用活中小微企业发展政策，支持各县区建设一批众创空间和星创天地，鼓励科研人员、大学生等各类群体创新创业。强化人才工作，抓好高层次紧缺人才的引进培养工作，年内引进50名急需的拔尖领军人才。

深化重点领域改革。加快行政体制改革，全面公布市县两级部门权力清单和责任清单，继续减少和规范行政许可审批事项，全面推行并联审批和网上审批；加快电子营业执照和企业登记全程电子化改革，健全完善不动产统一登记制度。稳步推进国有企业改革，开展市属国有企业股份制改革和国有资产证券化改革，推行市属经营性国有资产集中统一监管。强化金融改革，支持发展各类产业基金，完善融资担保体系，引进银行、保险、证券等金融机构，支持民间资本参与设立村镇银行，建立多层次的资本市场。加快农村产权制度改革，全面完成农村土地承包经营权确权登记颁证工作，建立集体产权交易市场，积极开展土地承包经营权抵押贷款业务，激活农村土地资本。

全面提升开放水平。深化国际交流合作，健全榆林海关通关联动机制，加快推进保税仓储、陆路口岸物流中心建设，推进贸易通关便利化；办好榆林国际煤炭暨能化装备技术博览会，支持外贸企业发展壮大，拓展榆林产品境外市场。加强区域经济合作，落实呼包银榆和陕甘宁区域合作定期协商机制，力争在基础设施互联互通方面取得新进展。强化招商引资工作，举全市之力瞄准重点领域、重点行业开展精准招商，力争落地一批重大新兴产业项目，确保全年招商引资到位资金600亿元。

（六）加强生态文明建设，推进绿色低碳发展。

狠抓生态环境建设。深入推进“生态建设五年行动计划”，继续实施“九大林业工程”和“五个百万亩基地”建设，全年完成造林绿化50万亩。推进环城防护林体系建设，打造榆林城区绿色生态屏障。抓好市境内的高速公路、国道、省道及地方道路两侧绿化工作。推进京津风沙源治理二期工程，开展沙柳等灌木平茬复壮工作。实施千村万户绿化工程，年内绿化美化村庄100个。稳步扩大经济林规模，发展海红果、长柄扁桃等经济林10万亩，实施红枣低产林改造10万亩。推进新一轮退耕还林工程，强化封山禁牧工作。做好自然保护区建设及湿地保护工作，争取红碱淖湿地列为国家自然保护区。

坚决整治环境污染。实施工业污染全面达标排放计划，抓好燃煤电厂超低排放改造工作，大力削减主要污染物排放总量。以面源和土壤重金属污染为重点，强化农村环境综合整治。实施无定河流域综合治理和榆阳河治理一期工程。切实加强水源地保护，确保饮用水水质安全。扎实推进煤矿采空塌陷区、火烧残留区治理，加强露天边角煤开发治理，做好境内输油管道改造提升工作。加大固体废物利用处置技术创新推广，加快陕北危险废物处置中心建设。全面加强环境保护监管，严格实行能源、水资源、建设用地总量和强度双控制度，推行自然资源资产离任审计，全面落实生态环境损害追究办法，从源头上防止污染环境、破坏生态。二氧化硫、氮氧化物分别削减4%，化学需氧量、氨氮分别削减2%。

倡导绿色生产和生活方式。抓好煤矿疏干水综合利用，提高城市生活污水集中处理率。提倡环保消费、适度消费，限制一次性用品使用和过度包装，启动生活垃圾分类投放、收集及资源化利用项目。支持合同能源管理实施节能改造，研究出台电能替代政策，推广电采暖、电锅炉等电气化设备。大力发展公共交通，增设公共交通停车港湾和公交站点，中心城区新开通4条公交线路。加快完善城市慢行系统建设，规划建设一批新能源汽车充电设施，增加一批绿色环保公交和出租车辆，引导居民低碳绿色出行。

（七）着力保障和改善民生，让老百姓得到更多实惠。

统筹发展各项社会事业。优先发展教育事业。深化教育领域综合改革，均衡配置区域、城乡教育资源，继续推进学校标准化建设和薄弱学校改造工程，建成高新三幼、高新六小和横山五中，启动29个公办幼儿园项目。加快中职资源整合，支持榆林学院升格榆林大学。加大对民办教育支持力度，提升民办教育水平。支持县区开展“教育强县”“双高双普”等创建工作，强化基层师资队伍建设，努力办好人民满意教育。加快发展卫生事业。深化公立医院综合改革，全面推行分级诊疗，加强基层医疗服务体系和人才队伍建设，抓好市级医疗机构专科建设，建成市关爱医院、传染病医院，支持神木县医院创三级、佳县和子洲县医院创二甲。全面落实两孩政策，提升人口计生服务水平。组织开展丰富多彩的文化惠民活动，大力弘扬社会主义核心价值观。启动建设榆林群众艺术馆、镇北台长城博物馆、榆林文化艺术中心，推进榆林博物馆、展览馆、图书馆等项目的前期工作，逐步补齐公共文化设施短板。积极发展体育事业，开展全民健身运动，启动榆林体育中心前期工作。支持国防和军队建设，不断提升军民融合发展水平。

提高社会保障能力。认真做好就业工作，多渠道、多方式开发就业岗位，加大职业技能培训力度，全年新增城镇就业2.8万人，转移农村劳动力67万人。实施全民参保计划，进一步扩大社会保险覆盖面，有序推进养老保险制度改革，落实五险一金精简归并政策，做好社会保障卡发行和应用工作，加快推进被征地农民参加社会养老保险的参保登记和养老待遇发放工作。完善残疾人社会保障制度，增强社区助残服务能力。加快完善社会救助体系，支持发展多种形式的养老服务机构。抓好保障性安居工程收尾工作，完善城市贫困人口的住房保障制度。做好农村留守妇女、儿童、老人的帮扶关爱工作，让他们生活得更加安心、省心、放心。

加强各类风险防控。努力防范金融风险，加强社会信用体系建设，依法打击非法集资、逃废金融债务和“老赖”行为；采取更加有效的措施化解民间借贷纠纷，加快案件审理和资产处置进程，切实维护群众合法权益。深化信访制度改革，健全多元化矛盾纠纷调解机制，畅通群众诉求表达渠道，妥善化解各类不稳定因素。加强食品药品监管，确保人民群众舌尖上的安全。做好社区矫正、法律援助、“七五”普法等工作，提高全社会的法治意识。严格落实安全生产责任制，坚决杜绝重特大事故发生。完善立体化社会治安防控体系，严厉打击暴力恐怖、涉黑涉恶犯罪。强化应急处置能力建设，完善各类应急机制，切实维护好人民群众的生命财产安全。

四、加强政府自身建设

适应新常态、谋求新发展、完成新任务，对政府自身建设提出了新的更高要求。我们必须认真践行“三严三实”，增强政治意识、大局意识，核心意识、看齐意识，全面加强和改进政府自身建设，不断提高政府治理现代化水平。

依法履职尽责。深入推进法治政府建设，健全依法决策机制，坚持重大决策合法性审查，推进行政机关法律顾问全覆盖，把政府工作纳入法治化轨道。依法接受市人大及其常委会的法律监督、工作监督，主动接受市政协的民主监督，广泛听取民主党派、工商联、无党派人士和各人民团体的意见，重视舆论和社会监督。加大政务公开力度，坚持开展邀请公民进政府活动，推广互联网+政务服务，努力打造运行规范、公开透明的阳光政府。

持续改进作风。认真开展“两学一做”学习教育，驰而不息纠正“四风”问题。探索建立合理的容错机制，把干部在推进改革发展中因缺乏经验而出现的无意过失和错误失误，与令行不止、明知故犯、以权谋私等违纪违法行为区分开来，鼓励干事，支持探索，最大限度地激发广大干部的干事创业热情。健全督查机制，加大问责问效，严肃整治不作为、慢作为和乱作为等行为，努力打造勤政担当、为民务实的效能政府。

加强廉政建设。始终把纪律和规矩挺在前面，从严落实党风廉政建设“一岗双责”，严格落实述职述廉、诫勉谈话、质询问责等制度，扎实推进惩治和预防腐败体系建设。前移审计、监察监督关口，强化政府采购、工程招投标、公共资源交易等领域的监管，从源头上预防腐败。严控论坛节庆活动，严控政府投资项目规模，严控政府一般性支出，确保“三公”经费只减不增，努力打造纪律严明、风清气正的廉洁政府。

各位代表，“十三五”发展的序幕已经拉开，榆林新的历史篇章等待着我们去书写。让我们紧密团结在以习近平同志为总书记的党中央周围，在省委、省政府和市委的坚强领导下，紧紧依靠全市广大干部群众，牢记使命，勇于担当，锐意进取，真抓实干，为率先全面建成小康社会而努力奋斗！
